# Parathyroid Hormone-Related Protein Is Not Required for Normal Ductal or Alveolar Development in the Post-Natal Mammary Gland

**DOI:** 10.1371/journal.pone.0027278

**Published:** 2011-11-08

**Authors:** Kata Boras-Granic, Joshua VanHouten, Minoti Hiremath, John Wysolmerski

**Affiliations:** 1 Department of Internal Medicine, Yale University School of Medicine, New Haven, Connecticut, United States of America; 2 Department of Biology, Boise State University, Boise, Idaho, United States of America; Baylor College of Medicine, United States of America

## Abstract

PTHrP is necessary for the formation of the embryonic mammary gland and, in its absence, the embryonic mammary bud fails to form the neonatal duct system. In addition, PTHrP is produced by the breast during lactation and contributes to the regulation of maternal calcium homeostasis during milk production. In this study, we examined the role of PTHrP during post-natal mammary development. Using a PTHrP-lacZ transgenic mouse, we surveyed the expression of PTHrP in the developing post-natal mouse mammary gland. We found that PTHrP expression is restricted to the basal cells of the gland during pubertal development and becomes expressed in milk secreting alveolar cells during pregnancy and lactation. Based on the previous findings that overexpression of PTHrP in cap and myoepithelial cells inhibited ductal elongation during puberty, we predicted that ablation of native PTHrP expression in the post-natal gland would result in accelerated ductal development. To address this hypothesis, we generated two conditional models of *PTHrP*-deficiency specifically targeted to the postnatal mammary gland. We used the MMTV-Cre transgene to ablate the floxed PTHrP gene in both luminal and myoepithelial cells and a tetracycline-regulated K14-tTA;tetO-Cre transgene to target PTHrP expression in just myoepithelial and cap cells. In both models of PTHrP ablation, we found that mammary development proceeds normally despite the absence of *PTHrP*. We conclude that PTHrP signaling is not required for normal ductal or alveolar development.

## Introduction

Mammary development begins during embryogenesis but is only completed during lactation. Morphogenesis occurs in stages: a rudimentary duct system is formed before birth; the ductal system is expanded during puberty; alveolar structures are formed during pregnancy; and full secretory differentiation is completed at the beginning of lactation [1], [2], [3], [4]. After weaning, the alveolar structures involute, but can redevelop with subsequent pregnancies to support repeated cycles of reproduction. Although mammary development is well described, the underlying molecular mechanisms that guide ductal and alveolar development are only partly understood [1], [2], [3], [4]. It has been well established that changes in the levels of circulating hormones initiate and guide each of the distinct stages of development noted above. Circulating ovarian and pituitary hormones act directly on mammary epithelial cells and also regulate local webs of growth factors and receptors to orchestrate epithelial-mesenchymal interactions critical to the integrated morphogenetic responses of the gland [2], [3], [5], [6]. These interactions between systemic hormones and local growth factor signaling networks are often deranged during the progression of breast cancers, so it is helpful to understand the normal effects of these systems during development in order to better understand how they go awry in tumors.

Before puberty, the murine mammary duct system consists of about 10–15 branches limited to that portion of the stromal fat pad nearest the nipple [2], [4], [6]. During puberty, under the influence of hormones, the ducts grow rapidly and directionally away from the nipple and through the fatty stroma, undergoing a process of ductal branching morphogenesis that eventually fills the entire fat pad with mature ducts, which serve as a scaffold upon which the alveoli form during pregnancy [2], [3], [6]. Ductal extension during puberty relies on the formation of highly proliferative, bulbous structures known as terminal end buds (TEBs) at the tips of the ducts. TEBs are composed of multiple epithelial cell layers surrounded by a fibrous stroma and adipocytes [3]. Ductal extension is completed by about 9 weeks of age, when the primary ducts have extended to the distal end of the mammary gland fat pad and the TEBs regress.

Parathyroid hormone-related protein (PTHrP) is a growth factor that binds to and activates a G protein–coupled receptor known as the Type 1 PTH/PTHrP receptor (PTHR1) [7]. Disruption of either gene in mice and loss of PTHR1 function in humans results in the absence of a mammary gland [7], [8], [9], [10], [11]. PTHrP is a product of normal embryonic mammary epithelial cells and the PTHR1 is found on immature mesenchymal cells located beneath the embryonic epidermis [8], [9], [10]. PTHrP is necessary for the formation of specific mammary mesenchyme and, in its absence, the embryonic mammary bud fails to form the neonatal duct system. PTHrP is also produced by mammary epithelial cells during lactation, where it circulates to increase bone resorption, liberating skeletal calcium stores that are used for milk production [12], [13].

The PTHrP gene is also expressed in the peripherally located epithelial cap cells of TEBs during puberty and the PTH1R is expressed in the stromal cells immediately surrounding TEBs [14], [15]. Overexpression of PTHrP in cap and myoepithelial cells using the keratin 14 (K14) promoter suggested that PTHrP acts in a paracrine fashion on stromal cells to slow the rate of ductal elongation during puberty [14]. PTHrP increased the basal rate of apoptosis among epithelial cells in TEBs and blocked the ability of estrogen to stimulate proliferation and decrease apoptosis in these cells [14]. These data suggested that PTHrP might act as an endogenous negative regulator of the effects of estrogen on TEBs. Herein, we report that PTHrP expression is found in the basal cells of the developing ducts and in the cap cells of terminal end buds during pubertal development. To address the physiological role of PTHrP in mammary development during puberty, we generated two conditional models of *PTHrP*-deficiency specifically targeted to the postnatal mammary gland. These mice undergo normal pubertal and pregnancy-associated mammary development despite the absence of PTHrP, suggesting that PTHrP signaling is not required for post-natal ductal and alveolar development.

## Results

### PTHrP expression during mammary gland development

**Figure 1:**
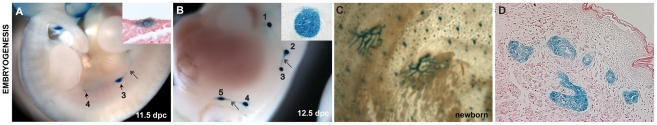
*PTHrP* expression during embryogenesis. LacZ staining of *PTHrP^+/^* ^lacZ^ embryos at (A) E11, (B) E12, (C) E15.5 and (D) birth. (A) At E11, β-galactosidase staining was observed in the mammary placodes but not the surrounding mesenchyme. (B) By E12.5, intense staining was observed in all five buds. Interestingly, lacZ positive “tails” were observed from each bud (double arrowhead) (B, C). Single arrowheads indicate mammary placodes and buds. (D) *PTHrP^lacZ^* expression remains restricted to the mammary epithelial cells throughout embryonic and neonatal development.

Embryonic deletion of PTHrP results in a lack of ductal outgrowth due to a failure in mammary mesenchyme specification [9]. At later stages of development, the functional role of PTHrP is not clearly defined. In order to begin to study the function of PTHrP during postnatal development, we first defined the pattern of *PTHrP* gene expression during all stages of mammary development. Transgenic mice in which the β-galactosidase gene has been knocked into the *PTHrP* locus provide a sensitive method for localizing endogenous PTHrP expression by staining for LacZ activity [16]. Using *PTHrP-lacZ* knockin mice (*PTHrP^lacZ^*), we defined *PTHrP* gene expression during embryogenesis, puberty, pregnancy and lactation. As shown in Figure 1A, lacZ expression was detected during mammary placode development in epithelial cells beginning at embryonic day 11.5 (E11.5). PTHrP expression also extended along a “tail” of epithelial tails adjacent to and sometimes between developing placodes within the mammary line. This pattern suggests that *PTHrP* gene expression is first activated in cells within the mammary line as they move towards the developing placodes and into the mammary buds. As embryonic development progresses, strong *PTHrP* expression remains restricted to the epithelial cells of the developing buds (Figure 1B), and the rudimentary ductal tree at birth (Figure 1C–D). Expression is absent in the adjacent stromal compartment at all stages of embryonic development.

**Figure 2:**
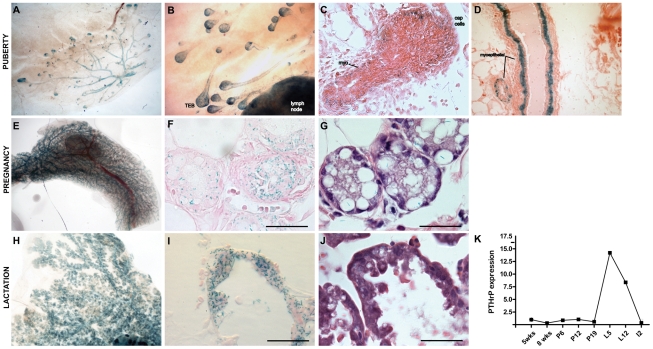
*PTHrP* expression during postnatal mammary gland development. (A) At the onset of puberty (3 weeks), *PTHrP^lacZ^* expression is seen throughout the ductal tree. (B) As development ensues, –mount Xgal staining is evident in the ducts and TEBs at 5 weeks, specifically in the myoepitheial cells and the cap cells (C). By 8 weeks, when TEBs have regressed, LacZ expression is restricted to myoepithelial cells in the ducts (D). During late pregnancy (E–G), LacZ expression is seen in the ducts and is also evident in the developing alveoli. During lactation (H–J), LacZ is expression is seen in the milk secreting cells. High levels of *PTHrP^lacZ^* staining remain in the ducts and the alveoli during lactation. (K) Developmental survey of PTHrP mRNA expression in whole mammary glands as measured by qRT-PCR. PTHrP mRNA is expressed at low levels in whole mammary glands throughout virgin postnatal development and throughout pregnancy. At the onset of lactation, PTHrP levels increase, and at involution return to virgin levels. wks = weeks; P = pregnancy day; L = lactation day; I = involution day. Relative expression: 5 weeks = 1. H&E staining (G, J).

Postnatal mammary glands are composed of two epithelial cell types, luminal and myoepithelial [6]. These two cell lineages form a bi-layered epithelium with the more centrally located luminal cells surrounded by a continuous layer of myoepithelial cells. The ducts are, in turn, surrounded by a few layers of peri-ductal fibroblasts and are embedded within a fatty stroma. Whole-mount analysis revealed *PTHrP^lacZ^* expression to be restricted to the epithelial cells within the mammary gland during puberty (Figure 2A–D). *PTHrP^lacZ^* expression was observed in the terminal end buds (TEBs) as well as the subtending ducts (Figure 2A–D). Histological sections demonstrated that *PTHrP^lacZ^* expression localized to the cap cells as well as to the monolayer of myoepithelial cells that line the entire duct system (Fig. 2D), confirming and extending our previous *in situ* hybidization data [15]. *PTHrP^lacZ^* expression was not detected in the body cells of the TEBs, the luminal cells of the ducts, the periductal fibroblasts, or the stromal adipocytes.

During pregnancy, the mammary epithelium expands dramatically as alveolar structures form at the end of small terminal ductules that develop from the pre-existing ducts [1], [3], [6]. Alveolar structures are specialized for milk production and it is thought that they arise from multipotent progenitor cells that can give rise to myoepithelial cells, ductal cells and alveolar cells [17]. During pregnancy, expression of *PTHrP* is seen in the myoepithelial cells of the ducts and in the developing alveolar cells and alveoli (Figure 2E–G). After pregnancy ends and lactation ensues, *PTHrP* expression is also evident in the milk secreting, alveolar epithelial cells (Figure 2H–J). It is well documented that these cells secrete large amounts of PTHrP into milk [12], [18]. The Xgal staining during postnatal development is consistent with RT-PCR data confirming that PTHrP mRNA is expressed at low levels throughout puberty and until the later stages of pregnancy. At the onset of lactation, levels increase and then return to baseline levels with involution (Figure 2K).

### Mammary-Specific Deletion of PTHrP Does Not Interfere with the Initial Stages of Mammary Ductal Outgrowth

**Figure 3:**
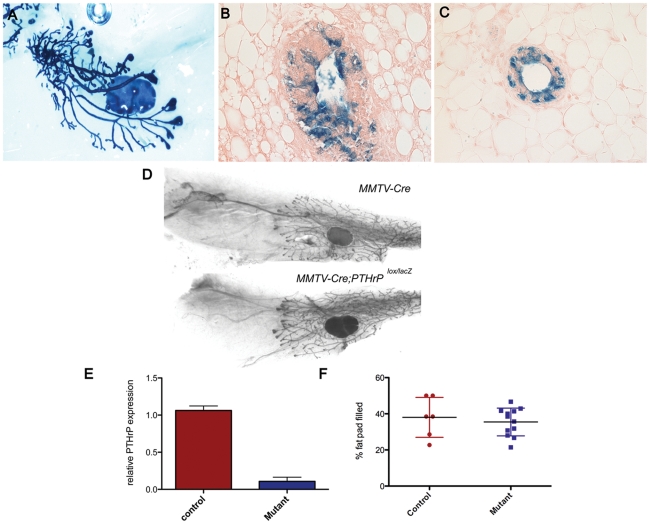
*MMTV-Cre* deletion of *PTHrP* in the mammary gland does not impair ductal development. (A) MMTV-Cre activity is heterogeneous in the pubertal gland. Whole-mount Xgal staining of a 5 week old gland. Sections of stained glands demonstrating that luminal and myoepithelial cells in the ducts (C), as well as body and cap cells of TEBs (B) are targeted for recombination. (D) Whole-mounts of mammary glands from *MMTV-cre* and *MMTV-cre;PTHrP^lox/lacZ^* mammary glands at 5 weeks of age. (E) *PTHrP* mRNA expression is decreased in *MMTV-cre;PTHrP^lox/lacZ^* mammary glands. (F) Ductal outgrowth was measured in Control (n = 6) and *MMTV-cre;PTHrP^lox/lacZ^* (n = 12) as % of fat pad filled.

Previous studies in our lab demonstrated that overexpression of PTHrP in myoepithelial cells inhibits ductal elongation during puberty by blocking the ability of estrogen to increase proliferation and decrease apoptosis in terminal end buds (TEBs) [14], [19]. In order to determine whether these results reflected the function of endogenous PTHrP during puberty, we disrupted PTHrP signaling during pubertal development. Given that *PTHrP^−/−^* animals die at birth and have no mammary glands [11], we conditionally deleted the *PTHrP* gene postnatally, after embryonic mammary development was completed. We used the MMTV-Cre, line D mice, which express Cre recombinase in both luminal epithelial and myoepithelial cells, including the body and cap cells of TEBs, beginning at the onset of puberty [20]. We confirmed transgene specificity by breeding MMTV-Cre mice to ROSA26R (R26R) mice (Figure 3A–C) [21]. β-galactosidase staining of whole mammary glands shows heterogeneous expression of the MMTV-*Cre* transgene during puberty, demonstrating that recombination is not 100% efficient [20]. Therefore, in order to maximally reduce local PTHrP levels, the MMTV-*Cre* transgene was bred onto a compound heterozygous *PTHrP^lox/lacZ^* background, with one *floxed PTHrP* allele and one *PTHrP^lacZ^* allele. *MMTV-Cre;PTHrP^lox/lacZ^* (MMTV-CKO) mice revealed that disruption of both *PTHrP* alleles had no effect on the initial mammary outgrowth (Figure 3D, and data not shown). Ductal extension was assessed morphometrically in MMTV-CKO and control mice (*MMTV-Cre* and *PTHrP^lacZ^*). As shown in Figure 3F, there were no significant differences in the degree of ductal extension of the glands. To ensure that the absence of a mammary gland phenotype was not due to the lack of efficient Cre-mediated *PTHrP* deletion in the mammary gland, we performed quantitative RT-PCR on whole mammary tissues from virgin control and MMTV-CKO animals (Figure 3E). These analyses demonstrated appropriate reduction of *PTHrP* mRNA in the MMTV-CKO mammary glands.

### Disruption of PTHrP in Myoepithelial Cells Does Not Alter Mammary Ductal Development

**Figure 4:**
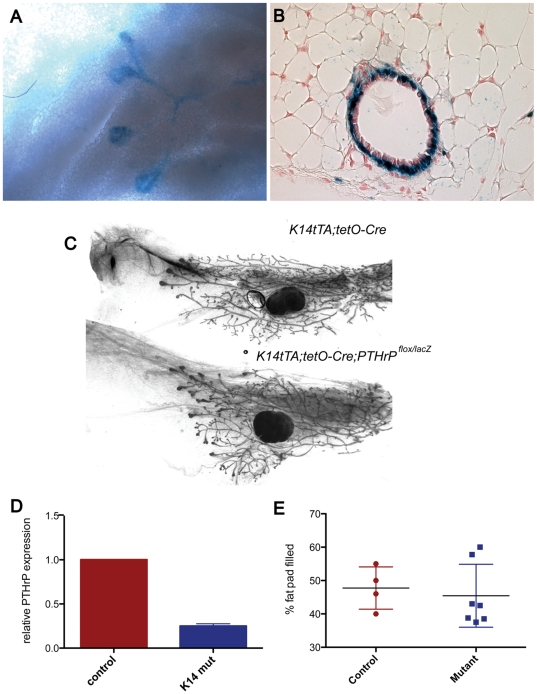
*K14-tTA;tetO-Cre* deletion of *PTHrP* in the mammary gland does not impair ductal development. *K14-tTA;tetO-Cre* activity is specific to the myoepithelial cells of the ducts and the cap cells of TEBs (A, B). (A) Whole-mount Xgal staining of a 4 week old gland. (B) Section of stained gland demonstrating that myoepithelial cells are targeted for recombination in the ducts. (C) Whole-mounts of mammary glands from *K14-tTA;tetO-Cre* and *K14-tTA;tetO-Cre;PTHrP^lox/lacZ^* mammary glands at 5 weeks of age. (E) *PTHrP* mRNA expression is decreased in *K14-tTA;tetO-Cre;PTHrP^lox/lacZ^* mammary glands. (F) Ductal outgrowth was measured in Control (n = 4) and *K14-tTA;tetO-Cre;PTHrP^lox/lacZ^* (n = 6) as % of fat pad filled.

Given our data demonstrating that PTHrP is expressed by myoepithelial cells and given the potential heterogeneous expression of the MMTV-Cre transgene in these cells during puberty, we also specifically deleted the *PTHrP* gene from myoepithelial cells in a temporally regulated fashion. Myoepithelial and luminal markers are both expressed in all embryonic mammary epithelial cells [9]. Therefore, in order to avoid targeting the embryonic mammary buds, we used a tetracycline-regulated system to initiate Cre expression in myoepithelial cells only after birth using bitransgenic *K14-tTA;tetO-Cre* mice (Figure 4A–B) to generate *K14-tTA;tetO-Cre;PTHrP^lox/lacZ^* mice (K14-CKO mice) [14]. The K14 promoter is strongly expressed in the basal cells of the gland and in the cap cells of TEBs, coinciding with the pattern of *PTHrP* expression as seen in Figure 2. Unlike the MMTV promoter, the K14 promoter drives uniform Cre recombinase expression in all myoepithelial cells during puberty as documented in *K14-tTA;tetO-Cre;R26R* mice (Figure 4A–B). As shown in Figure 4D, *PTHrP* mRNA expression was efficiently reduced in the mammary glands using this strategy. However, as with the previous approach, whole-mount analysis from K14-CKO animals revealed that disruption of one or both *PTHrP* alleles had no effect on the initial mammary outgrowth (Figure 4C, and data not shown). Ductal extension during puberty was normal in the K14-CKO mice (Figure 4C&E).

### Ablation of Endogenous PTHrP has no Effect on TEB Cell Turnover

**Figure 5:**
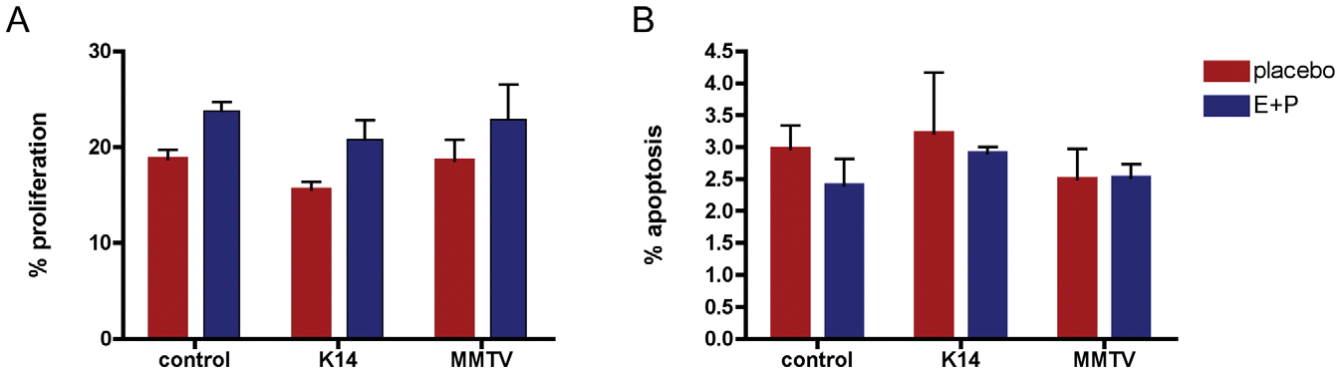
Effects of *PTHrP* deletion on cell turnover in terminal end buds during puberty. (A) End bud cell proliferation in 5-week-old mice as defined by the percentage of epithelial cells incorporating EdU within TEBs of control, *K14-tTA;tetO-Cre;PTHrP^lox/lacZ^* and *MMTV Cre;PTHrP^lox/lacZ^* mice. (B) Apoptosis was measured by TUNEL staining in the TEBs of control, *K14-tTA;tetO-Cre;PTHrP^lox/lacZ^* and *MMTV Cre;PTHrP^lox/lacZ^* mice. Red bars represent the baseline rates of proliferation and apoptosis in 5-week-old placebo-treated mice and blue bars represent rates of proliferation and apoptosis in 5-week-old mice treated with exogenous estradiol and progesterone for 48 h. EdU incorporation was significantly greater in each group of mice treated with hormones. There were no differences in the response to hormones in control mice as compared to the two types of CKO mice. There were no differences in apoptosis among control or CKO mice at baseline versus treated with hormones.

Previous experiments had demonstrated that overexpression of PTHrP in cap and myoepithelial cells blunted the effects of exogenous hormones on cellular proliferation and apoptosis within the TEBs, raising the possibility that endogenous PTHrP might normally act to modulate the effects of steroid hormones on TEBs during puberty [14]. Therefore, we predicted reciprocal changes in TEB cell turnover in the absence of *PTHrP* expression (i.e. enhanced proliferation and reduced apoptosis). We studied mice during early puberty (5 weeks of age) at baseline and after 48 h of treatment with exogenous estrogen and progesterone. Cell proliferation and apoptosis were quantified using EdU (5-ethynyl-2′-deoxyuridine) incorporation and TUNEL staining, respectively (Figure 5 and data not shown). At baseline, 18% of epithelial cells in wild-type TEBs incorporated EdU. Hormone treatment increased TEB proliferation such that 24% of epithelial cells within TEBs were labeled. As shown in Figure 5A, ablation of PTHrP did not affect either baseline or hormone-treated rates of proliferation within the TEBs; there were no differences between wild-type mice and MMTV-CKO or K14-CKO mutants. Similar results were seen for measurements of apoptosis (Figure 5B). In TEBs from WT mice, 3% of epithelial cells were TUNEL-positive at baseline and 2.4% of the cells were TUNEL-positive after hormone treatment. There were no significant differences in the rate of apoptosis in TEBs at baseline or in response to hormone treatment in either MMTV-CKO or K14-CKO mice as compared to controls.

### Ablation of Endogenous PTHrP has no Effect on Mammary Epithelial Cell Lineage

**Figure 6:**
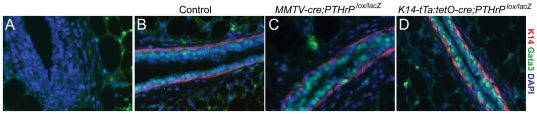
Cell lineage is not disrupted by *PTHrP* deletion in the mammary gland. Expression of the luminal marker, Gata3 and myoepithelial marker, K14, showed normal mammary gland architecture in both *PTHrP* MMTV-CKO (C) and K14-CKO (D) as compared to controls (B). (A) IgG control staining with secondary antibodies.

Deletion of the *PTHrP* gene from embryonic mammary epithelial cells results in reversion of these cells to an epidermal phenotype. Given the specific expression of *PTHrP* in myoepithelial cells, we next asked whether PTHrP was needed to maintain the basal lineage in postnatal epithelial ducts. In order to assess lineage specification, we stained mammary glands in both PTHrP conditional mutants using antibodies against K14 and p63, which recognize cap cells and myoepithelial cells [14], [22], and gata3 and K18, which identify body and ductal luminal cells [23]. As shown in Figure 6 (and data not shown), loss of PTHrP did not appear to affect the overall bilayered architecture of the ducts or the relative locations of luminal and myoepithelial cells in either conditional knockout model.

### PTHrP is not required for Terminal Differentiation of Alveolar Epithelial Cells

**Figure 7:**
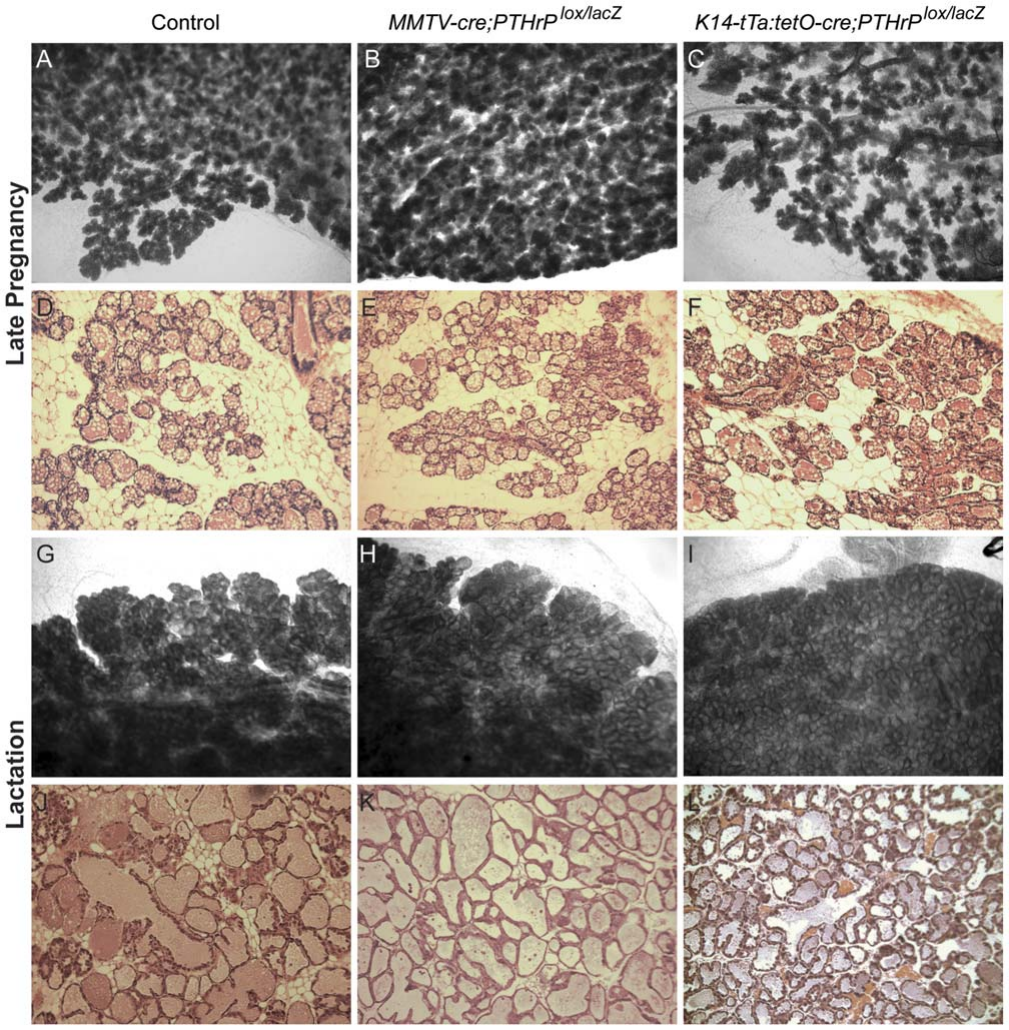
Loss of *PTHrP* has no Effect on Alveolar Development. Whole-mount analysis and histological H&E sections of control (A, D, G, J), MMTV-CKO (B,E,H,K) and K14-CKO (C,F,I,L) mice during late pregnancy (A–F) and lactation (G–L).

The *PTHrP* gene is expressed during pregnancy in alveolar epithelial cells (Figure 2E). We have previously demonstrated that *PTHrP* deletion during late pregnancy and lactation had no effect on alveolar terminal differentiation [12]. Nevertheless, we next examined whether deletion of *PTHrP* during pubertal development might affect alveolar development. Whole-mount analysis and histological examination revealed normal alveolar development on day 18 of pregnancy in both conditional knockout models of PTHrP deficiency (Figure 7A–F). In addition, histological analysis of lactating glands demonstrated normal alveolar differentiation with milk production. Both conditional mutant strains were able to sustain pups throughout lactation. We did not observe any weight differences between pups from MMTV-CKO, K14-CKO or control mothers (data not shown). Finally, no detectable defects in alveolar development were present even after multiple round of pregnancy and lactation.

## Discussion

PTHrP has important functions during embryonic breast development and during lactation. In the embryo, PTHrP is produced by mammary epithelial cells and it interacts with the PTHR1 on surrounding mesenchymal cells to support proper mammary mesenchyme differentiation and outgrowth of the mammary bud [4], [9], [11]. During lactation, PTHrP is produced by mammary epithelial cells and is secreted both into milk and into the maternal circulation, where it regulates systemic calcium and bone metabolism during lactation and contributes to the mobilization of calcium for milk production [12], [13]. PTHrP also contributes to the pathophysiology of breast cancer through its actions on tumor cells themselves, on the tumor microenvironment and on bone cells to promote the development of osteolytic bone metastases [24], [25], [26], [27]. Thus, PTHrP is intimately involved in the regulation of breast development, breast physiology and breast cancer pathophysiology. In this report, we examined whether PTHrP is also important for the development of the postnatal mammary gland.

We first examined the expression of *PTHrP*. Previous studies using *in situ* hybridization had suggested that *PTHrP* was expressed within the cap cells of TEBs during puberty [15]. For the current studies, we made use of a mouse in which the β-galactosidase gene was knocked into the *PTHrP* gene locus in order to improve the sensitivity and spatial resolution of detecting *PTHrP* expression [16]. Importantly, our studies confirm the expression of *PTHrP* within epithelial cells of the embryonic mammary bud as well as demonstrate *PTHrP* gene expression within segments of the mammary line, especially between buds 2 and 3, and buds 4 and 5. We confirmed that *PTHrP* is expressed within cap cells of the TEBs, but also now demonstrate that *PTHrP* is expressed within the mature myoepithelial cells in the adult mammary gland during puberty and throughout the reproductive cycle. These findings are consistent with previous reports of *PTHrP* expression exclusively in basal cells isolated from both human and mouse mammary glands [28], [29]. The *PTHrP* gene is not expressed in luminal or alveolar epithelial cells until pregnancy, when *lacZ* expression was detected within the developing alveolar epithelial cells. Importantly, *PTHrP* is expressed only in mammary epithelial cells; we did not detect its expression in stromal cells within the mammary fat pad at any time. These data demonstrate that, after embryogenesis, the *PTHrP* gene is expressed only by myoepithelial/cap cells until pregnancy and lactation, when alveolar epithelial cells also produce PTHrP.

The expression of *PTHrP* in cap and myoepithelial cells in close proximity to the expression of the PTHR1, which is expressed on stromal cells, suggests that PTHrP might regulate epithelial/mesenchymal interactions important for ductal extension and branching. In fact, overexpressing PTHrP in cap and myoepithelial cells using the keratin 14 promoter impaired ductal elongation by altering TEB cell proliferation and apoptosis in response to estrogen and progesterone [14], [30]. Overexpression of PTHrP also impaired ductal side-branching in this model [14], [30]. Therefore, we expected that disruption of the PTHrP gene in mammary epithelial cells would result in a reciprocal phenotype of accelerated ductal elongation and increased ductal side-branching. In contrast, *MMTV-Cre/PTHrP^lox/lacZ^* mice had normal rates of ductal elongation and a normal mammary ductal branching pattern. Additionally, they had normal rates of TEB proliferation and apoptosis, both at baseline and in response to exogenous hormones. This was also the case when we disrupted the PTHrP gene specifically within cap and myoepithelial cells using tetracycline-regulated bitransgenic expression of Cre recombinase. *K14-tTA;tetO-Cre;PTHrP^lox/lacZ^* mice also had normal ductal extension and branching as well as normal TEB cell proliferation and apoptosis. Both models of conditional *PTHrP* gene disruption also displayed normal alveolar development during pregnancy and both types of mice lactated normally (data not shown). Therefore, it appears that *PTHrP* expression is not necessary for post-natal mammary development.

It is not clear why disruption of the PTHrP gene had no effect on development of the gland given the effects of PTHrP overexpression on ductal morphogenesis. There are two main possibilities. First, it is possible that the spatial or temporal pattern of *PTHrP* gene disruption was insufficient to eliminate PTHrP function entirely. While the expression of Cre is somewhat heterogeneous in the MMTV-Cre mice, the expression of Cre in myoepithelial and cap cells is very uniform as measured by the ability to activate lacZ expression when these transgenes were bred onto an R26R reporter mouse (see Figure 4). Furthermore, RT-PCR analysis of *PTHrP* mRNA expression in whole mammary glands documented effective reductions of PTHrP mRNA expression (Figure 3E and 4D). Thus, a technical explanation for our findings is unlikely. Instead, we believe that while PTHrP contributes to the regulation of ductal morphogenesis, its functions are likely redundant and other signaling systems are able to compensate for its loss. This does not necessarily mean that PTHrP is unimportant in mammary epithelial cells or that the K14-PTHrP overexpression phenotype is an artifact. In fact, since PTHrP levels are often upregulated in breast cancer cells, the results of PTHrP overexpression in the mammary gland may shed light on its effects on tumor cells. In various studies, a higher level of PTHrP expression has been shown to be associated with either worse or better outcome in patients with breast cancer [31], [32]. Likewise, PTHrP appears to inhibit tumor formation in the MMTV-Neu transgenic model [26], but to promote tumor formation in the MMTV-polyoma middle T (MMTV-PyMT) transgenic model [33]. Therefore, depending on the context, upregulation of PTHrP expression may have differing effects on the biology of breast cancer. While normal epithelial cells do not express the PTHR1 gene, breast cancer cells often do. In addition, PTHrP can traffic to the nucleus and exert effects on cell proliferation and survival [27], [34]. As a result, autocrine and/or intracrine PTHrP signaling may affect breast cells in different ways depending on cell lineage or stage of transformation. Overexpression models will be necessary to sort out these issues.

In summary, we find that the *PTHrP* gene is expressed in myoepithelial and cap cells during post-natal mammary development. During pregnancy and lactation, the gene is also expressed by the secretory alveolar cells. Disruption of the *PTHrP* gene in all mammary epithelial cells using the *MMTV-Cre* transgene or only in myoepithelial and cap cells using a bitransgenic *K14-tTA;tetO-Cre* system, results in no discernable developmental phenotype. We conclude that PTHrP signaling is not required for post-natal mammary development.

## Materials and Methods

### Mouse strains and breeding

*MMTV-Cre* (line D), *tetO-Cre* and *ROSA26R* mice were purchased from Jackson Laboratories [20]. *K14-tTA* transgenic mice were previously generated in our laboratory [14]. *PTHrP^lacZ^* knockin mice were a kind gift from Arthur Broadus [16]. *PTHrP^lox/lox^* mice have been described elsewhere [35]. All animals were outcrossed more than 10 generations and maintained on a CD1 background (Charles River Laboratories, Wilmington, Massachusetts, USA). *TetO-Cre* transgene expression was suppressed by feeding all pregnant bitransgenic mice 150 µg/ml tetracycline hydrochloride (Roche, Indianapolis, IN, USA) in 5% sucrose water until birth. All animal experimentation was conducted in accord with accepted standards of humane animal care and approved by the Yale IACUC. All experiments performed were approved in advance by Yale University's Institutional Animal Care and Use Committee (protocol #07834).

### ß-Galactosidase assay

ß-galactosidase activity was measured in embryonic and adult mammary tissue as previously described [14].

### RNA isolation and RT-PCR

Mammary glands were excised from mice, flash frozen in liquid nitrogen and total RNA was isolated using TRIzol (Invitrogen, Carlsbad, CA) as per the manufacturers' instructions. Contaminating DNA was removed using the RNeasy Minikit and DNase 1 treatment (QIAGEN, Inc., Valencia, CA). Two-step quantitative real-time-PCR was performed using the High Capacity cDNA archive kit (Applied Biosystems, Foster City, CA) and the Full-Velocity SYBR-Green QPCR Master Mix kit (Stratagene, La Jolla, CA) in the Opticon 2 DNA Engine (MJ Research, Waltham, MA). The relative expression levels were determined using the comparative 2^−ΔΔCT^ method. Glyceraldehyde-3-phosphate dehydrogenase was the endogenous reference gene, and the average 2^−ΔΔCT^ of the samples from virgin mice served as a calibrator sample to which all individual samples were normalized. Each sample was run in triplicate. The following primers were used: mouse glyceraldehyde-3-phosphate dehydrogenase, forward, 5′-CGTCCCGTAGACAAAAATGGT-3′ and reverse, 5′-TCAATGAAGGGGTCGTTGAT-3′; mouse PTHrP, forward 5′-TTCAGCAGTGGAGTGTCCTG-3′ and reverse, 5′-TTGCCCTTGTCATGCAGTAG-3′.

### Whole-mount analysis and Tissue Processing

Whole-mount analysis was performed on mammary tissue as previously described [19]. Briefly, the no. 4 inguinal mammary glands were removed and mounted on a microscope slide. The tissue was fixed in acid ethanol for 1 h at room temperature, washed in 70% ethanol and distilled water and incubated in carmine aluminum stain (0.2% carmine, 0.5% aluminum potassium sulfate) overnight at room temperature. After staining, the mammary glands were dehydrated through graded ethanol and defatted in acetone and toluene before being mounted under glass coverslips using Permount (Fisher Scientific, Fair Lawn, NJ, USA). Histomorphometry was performed on whole mounts and the following parameters were measured: total duct length was measured from the nipple region to the leading edge of the most distal end bud; the percentage of fat pad penetration was calculated by dividing the total duct length by the distance from the nipple region to the most distal aspect of the fatty stroma.

For light microscopy, mammary tissue was fixed in 4% paraformaldehyde, embedded in paraffin and sectioned at 5 µm for hematoxylin & eosin (H&E) staining or immunohistochemistry.

### Hormone Treatment and Immunohistochemistry

Five-week-old mice were injected (i.p.) with an aqueous solution of estradiol (24 µg/day) and progesterone (1.2 mg/day) for 2 days before injection of EdU. Control mice were injected with PBS [19]. EdU (Invitrogen) was injected 2 hrs before the animals were sacrificed, after which the no. 4 inguinal mammary glands were harvested, fixed in 4% paraformaldehyde overnight, washed in 70% ethanol, embedded in paraffin and sectioned at 5 µm. The sections were then deparaffinized, rehydrated through graded ethanol to distilled water and put in pressure cooker for 1 minute in 10 mM sodium citrate buffer. Sections were then processed for EdU Click-It and immunofluorescence for the lineage markers, K14, p63, gata3, K18. Fifteen TEBs from a total of five mice per genotype were counted for each experimental condition. Rates of TEB proliferation and apoptosis were calculated by dividing the number of EdU positive nuclei by the total number of nuclei within five TEBs from each of five animals for each genotype.

### Statistics

The differences of the mean values between *PTHrP* mutant and control groups were compared using an unpaired *t* test. All *t* tests were two-tailed. All statistical analyses were carried out using Graph Pad Prism 4.0 for Windows (Graph-Pad Software, San Diego, CA).
